# Physiological mechanisms contributing to increased water-use efficiency in winter wheat under organic fertilization

**DOI:** 10.1371/journal.pone.0180205

**Published:** 2017-06-29

**Authors:** Linlin Wang, Shiwen Wang, Wei Chen, Hongbing Li, Xiping Deng

**Affiliations:** 1 State Key Laboratory of Soil Erosion and Dryland Farming on the Loess Plateau, Northwest A&F University, Yangling, Shaanxi, China; 2 College of Life Sciences, Northwest A&F University, Yangling, Shaanxi, China; 3 Key Laboratory of Disaster Monitoring and Mechanism Simulating of Shaanxi Province, Baoji University of Arts and Sciences, Baoji, Shaanxi, China; Institute of Genetics and Developmental Biology Chinese Academy of Sciences, CHINA

## Abstract

Improving the efficiency of resource utilization has received increasing research attention in recent years. In this study, we explored the potential physiological mechanisms underlying improved grain yield and water-use efficiency of winter wheat (*Triticum aestivum* L.) following organic fertilizer application. Two wheat cultivars, ChangHan58 (CH58) and XiNong9871 (XN9871), were grown under the same nitrogen (N) fertilizer rate (urea-N, CK; and manure plus urea-N, M) and under two watering regimes (WW, well-watered; and WS, water stress) imposed after anthesis. The M fertilizer treatment had a higher P_n_ and lower g_s_ and T_r_ than CK under both water conditions, in particular, it significantly increased WRC and Ψ_w_, and decreased EWLR and MDA under WS. Also, the M treatment increased post-anthesis N uptake by 81.4 and 16.4% under WS and WW, thus increasing post-anthesis photosynthetic capacity and delaying leaf senescence. Consequently, the M treatment increased post-anthesis DM accumulation under WS and WW by 51.5 and 29.6%, WUE_B_ by 44.5 and 50.9%, grain number per plant by 11.5 and 12.2% and 1000-grain weight by 7.3 and 3.6%, respectively, compared with CK. The grain yield under M treatment increased by 23 and 15%, and water use efficiency (WUE_g_) by 25 and 23%, respectively. The increased WUE under organic fertilizer treatment was due to elevated photosynthesis and decreased T_r_ and g_s_. Our results suggest that the organic fertilizer treatment enabled plants to use water more efficiently under drought stress.

## Introduction

Water shortage is the main abiotic factor limiting plant production on the Loess Plateau of China, with crop growth and economic yield being severely affected. Although the vegetative growth of winter wheat in the area occurs during relatively good soil moisture conditions, the grain filling is often impaired by terminal water stress. In most cases, crops may not be able to use nitrogen (N) efficiently if water is a limiting factor for growth and production [1]; thus, N content is also a limiting factor for crop production in dryland cropping systems [2]. To achieve higher crop yield, the application of synthetic N fertilizers has markedly increased in recent years [3]. The increasing use of inorganic fertilizer and neglect of organic fertilizer as a valuable source of nutrients have contributed to nutrient imbalance, low fertilizer use efficiency [4], deterioration in soil quality [5–7], nitrate leaching, and nitrous oxide emissions, soil acidification [8] and carbon (C) loss [9], which seriously limit crop productivity. Therefore, the effective use of limited water resources and chemical fertilizer to achieve high yield is a major objective of modern agriculture in the Loess Plateau, and will have a considerable impact at local and regional scales.

The effects of fertilizers on wheat yield and water use efficiency (WUE) have been intensively studied. Generally, the application of inorganic fertilizer can increase crop yield by increasing biomass accumulation, but greater biomass accumulation increases transpirational leaf area, creating excessive transpiration and water loss from the crop canopy, which in turn cause severe soil water depletion during the wheat-growing season in semi-arid regions [10–12]. Thus, in the long run, increased N fertilization may not be sufficient to maintain high yields and WUE due to continual soil water depletion [13,14]. However, a number of studies have shown that organic fertilizer input could increase the soil water-holding capacity [10,13] and successfully match N availability with crop uptake [14], thereby improving yield and WUE. Liu et al. [15] found that organic fertilizer increased the soil water-holding capacity by increasing the percentage of macro-aggregates (> 0.25 mm) and soil nutrients, which ultimately improved yield and WUE. Most importantly, the significant increases in WUE under organic fertilizer treatment was not a function of higher water uptake [10,13,14]. Although some earlier studies have explored the effects of organic fertilizer application on yield and WUE, they mainly focused on changes in soil water storage and the water-holding capacity; there is little understanding of the physiological basis of improved WUE following organic fertilizer application.

Generally, to our knowledge, there is no uniform view on the physiological mechanism by which winter wheat regulates WUE. Some studies have reported that increased plant drought tolerance and decreased leaf water loss, which can reduce damage to plants and enable them to maintain higher net photosynthetic rate (P_n_), play an important role in improving WUE [4,16]. There are some reports that root pruning increases yield and WUE by maintaining a higher P_n_ and reducing transpiration (T_r_) and stomatal conductance (g_s_) [17,18]. These reports suggest that maintaining a higher P_n_ and reducing water loss from the leaf surface (i.e., effective use of water) are essential for maximizing WUE. The carbohydrates for grain filling in wheat mainly originate from respective contemporaneous leaf and ear photosynthesis [19,20] and mobilization from source organs during grain filling [21]. Therefore, dry matter (DM) accumulation and remobilization during grain filling are important processes for yield formation and WUE. However, information on the effects of organic fertilizer on the DM accumulation and remobilization is scanty. Therefore, the objectives of this study were to evaluate the effect of organic fertilizer on (i) the response of winter wheat to water stress based on leaf relative water content (LRWC), malondialdehyde (MDA) content, activities of superoxide dismutase (SOD) and peroxidase (POD), and photosynthetic characteristics, (ii) DM accumulation and remobilization, and (iii) water use efficiency. The results are expected to improve the theoretical underpinning of high-yield and high-efficiency cultivation of winter wheat.

## Materials and methods

### Plant material and experimental design

A pot experiment was conducted to assess the effects of organic fertilizer on grain yield and WUE of wheat in Yangling, Shaanxi Province, in northwest China (34°16′N, 108°4′E; 460 m above sea level), using plastic pots (30 cm in diameter × 33 cm high) filled with 12 kg of sieved topsoil (26% soil water capacity, SWC). Soil collection was permitted by the land owner and we ensured that it did not involve endangered or protected species. The soil was Eum-Orthrosols (Chinese soil Taxonomy) and contained 0.87 g kg^−1^ total N and 10.0 g kg^−1^ organic matter. The readily available P and K concentrations were 16.45, and 117.64 mg kg^−1^, respectively. A movable shelter was used to prevent wetness during rainy days. Two different drought resistance wheat varieties, cv. ChangHan 58 (CH58) and XiNong9871 (XN9871) were studied, the former having a stronger drought resistance than the latter, 84 pots were used in this experiment for each wheat variety, twenty-four seeds of winter wheat were sown per pot at a depth of 5 cm on 15 October and thinned to 12 seedlings per pot on 30 October. After thinning, the soil in the pots was covered with perlite to reduce evaporation. Plants received the identical N level (2.8 g urea-N per pot, CK; and 1.4 g urea-N plus 180 g dried cattle manure per pot, M). Organic N sources were applied at target rate of 1.4 g pot^–1^ total N, considering that on average the organic N sources contain 80% available N [22]. The manure contained total N, P and available K of 0.97%, 0.45% and 6.22 g kg^–1^, respectively. Phosphorus and potassium (K) were applied at 4 g of P_2_O_5_ and 3 g of K_2_O per pot before sowing using triple superphosphate and potassium sulfate. All the urea-N fertilizer was applied as sowing. Hand weeding was used throughout the growing period; no incidence of disease or pest was found during experimentation.

The plants were irrigated to 70–80% SWC before anthesis. Drought stress (40–50% SWC) was introduced by reducing the water supply during the grain-filling period. The wheat plants were irrigated every day to 70–80% SWC for well-watered plants (WW) and 40–50% SWC for water stressed plants (WS) by weighing during the grain-filling stage. The two wheat cultivars have different periods of anthesis (defined as when 50% of the heads in a pot flower). For XN9871, anthesis occurred on 23 April and harvest on 28 May; for CH58, anthesis was on 29 April and harvest on 3 June.

### Weather conditions

**Fig 1:**
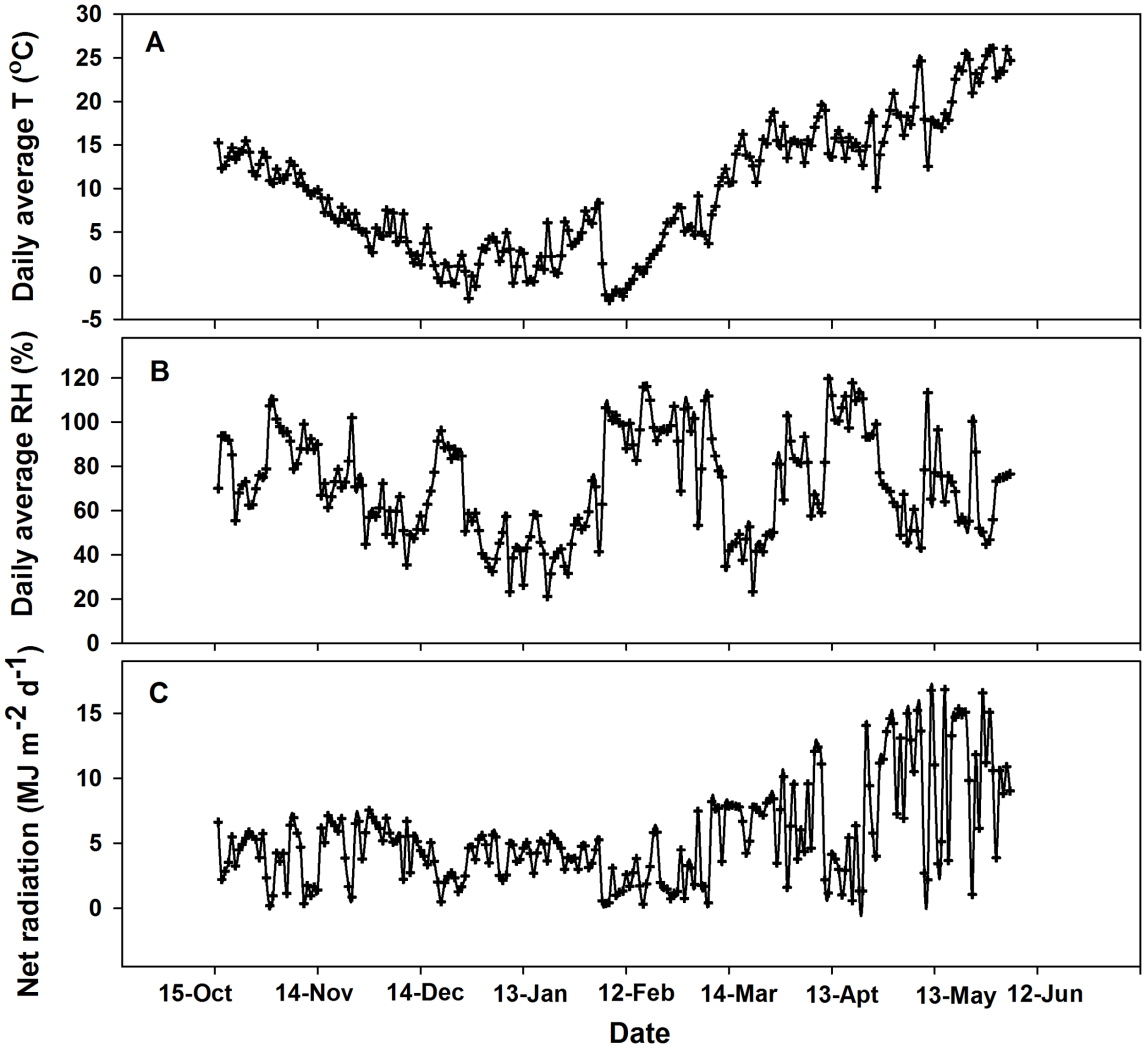
Diurnal temperature (T), relative humidity (RH) and net radiation during the growing seasons of winter wheat.

Weather data from an automatic weather station approximately 150 m from the experimental site were used. The key weather parameters measured were temperature, humidity, wind speed, radiation and rainfall at hourly intervals. The daily average temperature (T), net radiation and relative humidity (RH) are shown in Fig 1.

### Measurement of water use efficiency

The total water consumption per plant during the grain-filling stage (TWC, kg plant^−1^), water consumption rate (WCR, g d^−1^ plant^−1^) during the grain-filling stage and WUE were calculated as follows:

$$\text{TWC}=\frac{\left[\text{TIA}+\left(\text{initial pot weight }-\text{ final pot weight}\right)\right]}{plant\;number}$$ (1)

$$\text{WCR}=\frac{\text{TWC}}{\text{D}}$$ (2)

$${\text{WUE}}_{\text{B}}\;=\frac{\text{PADMA}´}{\text{TWC}}$$ (3)

$${\text{WUE}}_{g}=\frac{\text{GY}}{\left({\text{TWC}}^{\text{'}}+\text{TWC}\right)}$$ (4)

Where initial pot weight is the pot weight at day 0 at anthesis, final pot weight is the pot weight at harvest, TIA is total irrigation amount during grain filling (kg pot^−1^), WUE_B_ is water use efficiency for biomass yield (g kg^−1^), PADMA´ is post-anthesis DM accumulation per plant (g plant^−1^), D is duration of grain filling (d), GY is grain yield (g plant^−1^), TWC´ is total water consumption from sowing to anthesis (kg plant^−1^) and WUE_g_ is water use efficiency for grain yield (g kg^−1^), respectively.

### Determination of photosynthetic rate and relative water content (RWC)

Net photosynthetic rate (P_n_), transpiration rate (T_r_) and stomatal conductance (g_s_) of flag leaf were measured using a LI-6400 portable photosynthesis system (LI-COR Inc., Lincoln, NE, USA) at 9:00–11:00 AM at 0-7-14-21-28 d after anthesis, the P_n_, T_r_ and g_s_ were measured with the same leaves every time, each treatment had six replications from three pots (each pot selected two plants). At the middle of grain filling (14 d after anthesis), six flag leaves from three pots were sampled at 8:00 AM and water potential was measured immediately. Water potential was measured by using a pressure chamber (Model 3500, Soil moisture Corp., Santa Barbara, CA, USA). Meanwhile, six flag leaves from three pots were sampled at 12:00 PM and fresh weight (FWt) and leaf area (LA) were measured immediately. Then, the saturated weight (SWt) was measured after soaking the leaves in distilled water (6 ± 2°C) in the dark for 24 h. Consequently, the saturated leaves were placed on a piece of plastic netting and weighed after 6 h (W6). Finally, the flag leaves were dried to measure dry weight (DWt). Leaf relative water content (LRWC), excised-leaf water loss rate (EWLR) and water retention capacity (WRC) were calculated as following [4,23]:

LRWC=(FWt − DWt)/(SWt − DWt) (5)

EWLR=(SWt − W6)/(LA × 6) (6)

WRC=(W6 − DWt)/(SWt − DWt) (7)

### Evaluation of the activity of antioxidative system enzymes and malondialdehyde (MDA) content

At the middle of grain filling (14 d after anthesis), six flag leaves from three pots were sampled and homogenized on ice with a mortar and pestle in a 0.1 M potassium phosphate buffer (pH 7.0). The homogenate was centrifuged at 12,000 g for 15 min at 4°C. The supernatant was used immediately for enzyme assays. The activity of SOD was measured using xanthine, xanthine oxidase, and cytochrome c [24]. One unit of SOD was defined as the amount of enzyme that inhibits the rate of ferricytochrome c reduction by 50%. The activity of POD was assayed according to the method described in Wang et al. [24], using pyrogallol as a substrate. One unit of POD activity was defined as the amount of enzyme necessary to obtain 1 mg of purpurogallin from pyrogallol in 20 s, at 420 nm. MDA content was measured using the modified thiobarbituric acid (TBA) method [24–26]. Specific absorbance of extracts was recorded at 532 nm. Non-specific absorbance at 600 nm was measured and subtracted from the 532 nm readings. The concentration of MDA was calculated as a measure of lipid peroxidation.

### Chlorophyll concentration (SPAD value) measurement

Chlorophyll contents were estimated non-destructively at the mid position of flag leaf blades using a SPAD 502 chlorophyll meter (Minolta, Japan) every 7 d till 28 d after anthesis. The same leaves were measured every time, each treatment had six replications from three pots.

### Maximum PSII quantum yield (Fv/Fm) measurement

Fv/Fm were determined using a pulse amplitude modulated chlorophyll fluorescence system (Imaging PAM, Walz, Effeltrich, Germany) every 7 d till 28 d after anthesis, according to the methods of Xu and Chen [27,28]. The flag leaves were dark-adapted for 30 min before measurement. Minimal fluorescence yield (Fo) was measured with relatively weak measuring light pulses (0.5 μmol m^−2^ s^−1^) at a low frequency (1 Hz), and maximal fluorescence yield (Fm) was determined by applying a pulse saturation light (duration 0.8 s, 1580 μmol m^−2^ s^−1^). The same leaves were measured every time, each treatment had six replications from three pots. The Fv/Fm [Fv/Fm = (Fm-Fo)/Fm] was calculated automatically using ImagingWin software (Version 2.40, Walz).

### Determination of dry matter and N accumulation and remobilization

At anthesis and maturity, three pots per treatment were used to measure dry matter and N accumulation and remobilization. The plant shoot was separated into lamina, stem (including sheath) and ear (grain and chaff at harvest). Roots were separated from the soil by washing with water until they were clean. Plant samples were dried at 80°C and then weighed. The dry vegetative samples were first ground in a hammer mill and then reground finely using a 1 mm screen for N analysis. Plant N content was determined by Kjeldahl method [29]. The following parameters, related to DM and N accumulation, remobilization and remobilization efficiency, were calculated following previous studies [30–33]:

- Post-anthesis DM accumulation (PADMA) = DM of roots, leaves, stems, grain and chaff at maturity—DM of the whole plant at anthesis;
- Post-anthesis N accumulation (PANU) = content of N in roots, leaves, stems, grain and chaff at maturity − content of N of the whole plant at anthesis;
- DM remobilization (DMR) = DM at anthesis − DM of roots, leaves, stems and chaff at maturity;
- DM remobilization efficiency = (DMR/DM of the whole plant at anthesis) × 100%.

### Assays of grain weight and grain filling rate

Twelve spikes from three plots every treatment were sampled every 3 d from flowering to 35 d after flowering to measure grain weight. A total of 180 sampled grains were used for measurements of grain dry weight and grain filling rate. These samples were dried at 70°C to constant weight and weighed. The processes of grain filling were fitted by Richards’ growth equation [34] as described by Zhang et al [35]:

W=A(1+Be−kt)1N (8)

Grain filling rate was calculated as the derivative of the Eq (8):

R=AkBe−ktN(1+Be−kt)(N+1)N (9)

where W is grain weight, A is the final grain weight, t is the time after anthesis (d), and B, k, and N are coefficients determined by regression. The grain filling period was defined as that when W was from 5% (t1) to 95% (t2) of A. The average grain filling rate during this period was calculated from t1 to t2 [35].

### Statistical analysis

Data were analyzed using SAS (Version 9.4 for Windows, SAS Institute, Inc., Cary, North Carolina) and SPASS software (Version 16.0 for Windows, SPSS, Chicago, USA) by performing analyses of variance for yield and physiological traits. Pearson correlation analyses in the SPSS System were used to assess correlations between final grain weight and the average grain filling rate during 14−28 d. A General Liner Model analysis of variance in the SPSS System was adopted. The effects of the fertilizer, cultivar, water and their interactions on the measured variables were evaluated using one- and three-way ANOVA. When F-values were significant, the least significant difference (LSD) test in the SAS System was used to compare means. The significant differences between the means were estimated at 95% confidence level.

## Results

### Effects of organic fertilizer on water relations

The organic fertilizer application (M treatment) had little effect on leaf relative water content (LRWC). Excised-leaf water-loss rate (EWLR) under both WW and WS conditions was significantly lower under M treatment than in CK. Water retention capacity (WRC) and leaf water potential (Ψw) under both WW and WS were higher in M treatment than in CK (Table 1). Under WS, the EWLR in M treatment was 19.4% lower than that in CK (*P*<0.05), whereas the WRC and leaf Ψw were 41.8 and 24.2% higher, respectively (*P*<0.05), than those in CK. The W × F interaction was statistically significant for Ψw, WRC and EWLR (*P*<0.05).

**Table 1:** Effects of organic fertilizer, water conditions and cultivars on leaf relative water content (LRWC), leaf water potential (Ψ_w_), excised-leaf water loss rate (EWLR), and water retention capacity (WRC) of two winter wheat cultivars.

Treatments			LRWC	ψ_w_	EWLR	WRC
Fertilizers (F)	Water (W)	Cultivars(C)		(MPa)	(mg cm^−2^ h^−1^)	
Control (CK)	WS	CH58	0.88	-2.39	7.20	0.56
		XN9871	0.88	-2.48	7.73	0.54
	WW	CH58	0.92	-1.30	9.67	0.68
		XN9871	0.92	-1.46	10.28	0.65
	Mean		0.90	-1.91	8.72	0.61
Organic fertilizer (M)	WS	CH58	0.91	-2.08	5.64	0.79
		XN9871	0.89	-2.15	6.39	0.77
	WW	CH58	0.95	-1.19	6.06	0.72
		XN9871	0.94	-1.28	6.76	0.70
	Mean		0.92	-1.68	6.21	0.75
LSD (0.05)			0.06	0.13	0.85	0.06
			Probability level of ANOVA			
W			**	***	***	ns
F			ns	**	**	***
C			ns	*	**	ns
W×F			ns	*	**	***
C×W			ns	ns	ns	ns
C×F			ns	ns	ns	ns
W×F×C			ns	ns	ns	ns

WS: water stress; WW: well-watered.

* indicated significant differences at *P* = 0.05 level

** indicated significant differences at *P* = 0.01 level

*** indicated significant differences at *P* = 0.001 level

ns: not significant.

### Effects of organic fertilizer on antioxidant enzyme activity and lipid peroxidation

**Fig 2:**
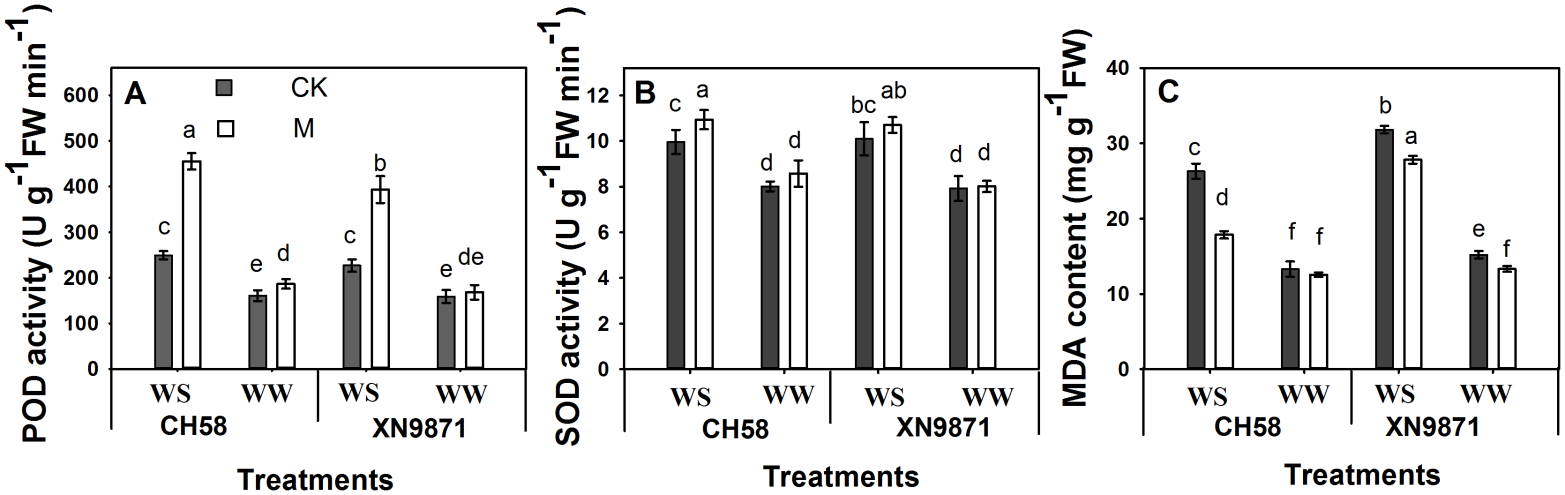
Effects of organic fertilizer on flag leaf (A) POD activity, (B) SOD activity and (C) MDA content of two wheat cultivars under water stress and well-watered conditions at 14 d after anthesis. CK: control; M: organic fertilizer; WS: water stress; WW: well-watered. All data were presented as the mean± SD of six replicates. Duncan’s multiple range test in the SPSS System was used to separate the means, different lowercase letters represent significant different (*P*≤0.05).

To investigate the differential oxidative damage between CK and M treatments under drought stress, we measured the POD and SOD activity and MDA content at 14 d after anthesis (Fig 2). MDA content and POD and SOD activity were significantly increased by WS (Fig 2A, 2B and 2C). However, the M treatment significantly increased POD activity and reduced MDA content compared with CK under WS (Fig 2A and 2C). Under WS, the SOD activity in CH58 was significantly higher in the M treatment than in CK, but no difference was observed for XN9871 (Fig 2B).

### Effects of organic fertilizer on maximum PSII quantum yield (Fv/Fm) and SPAD of flag leaf

**Fig 3:**
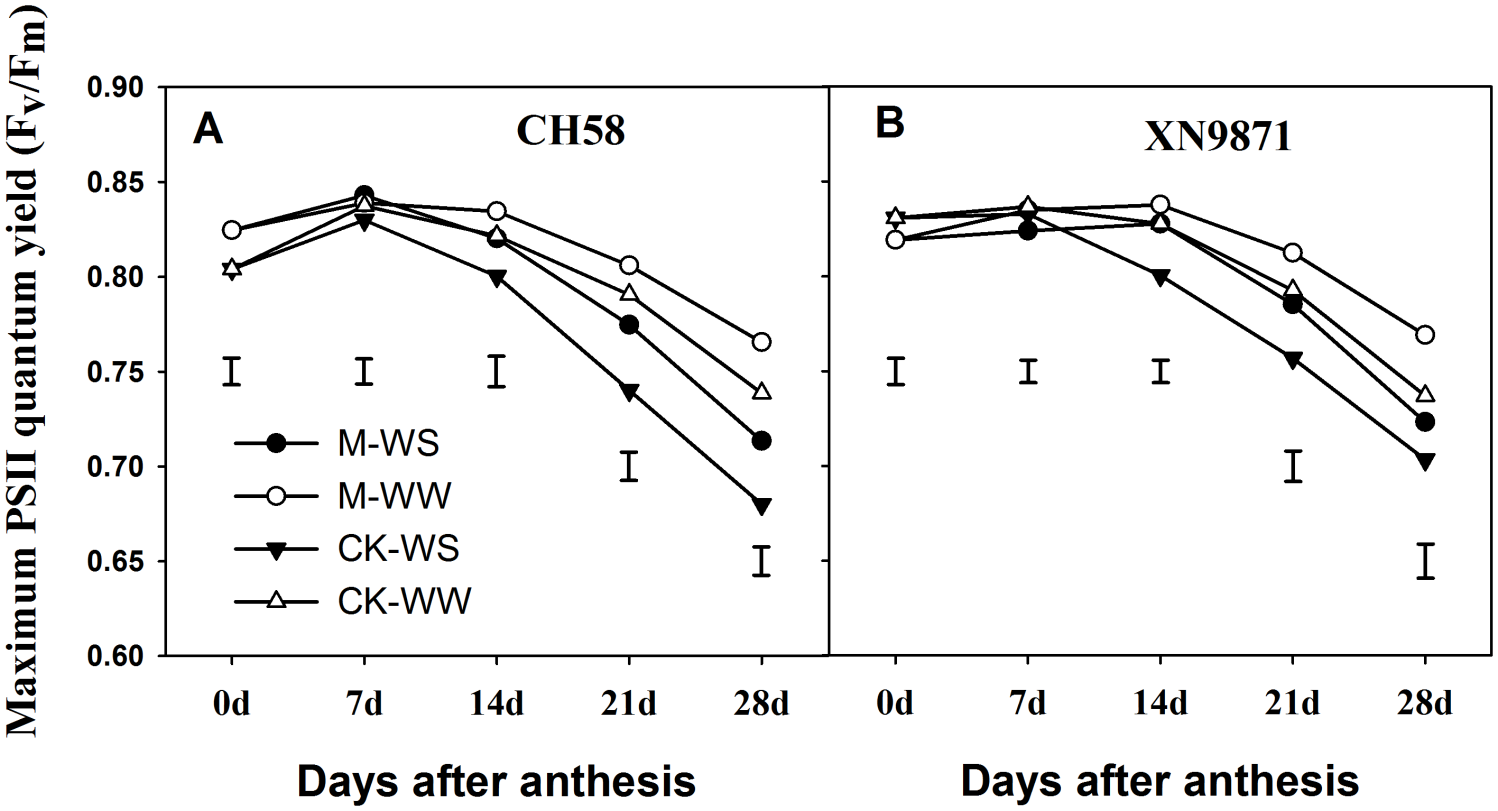
Changes in Fv/Fm of the flag leaf of two wheat cultivars, CH58 (A) and XN9871 (B) under organic fertilizer and different water regimes. CK: control; M: organic fertilizer; WS: water stress; WW: well-watered. Bars are LSD at *P*≤0.05.

**Fig 4:**
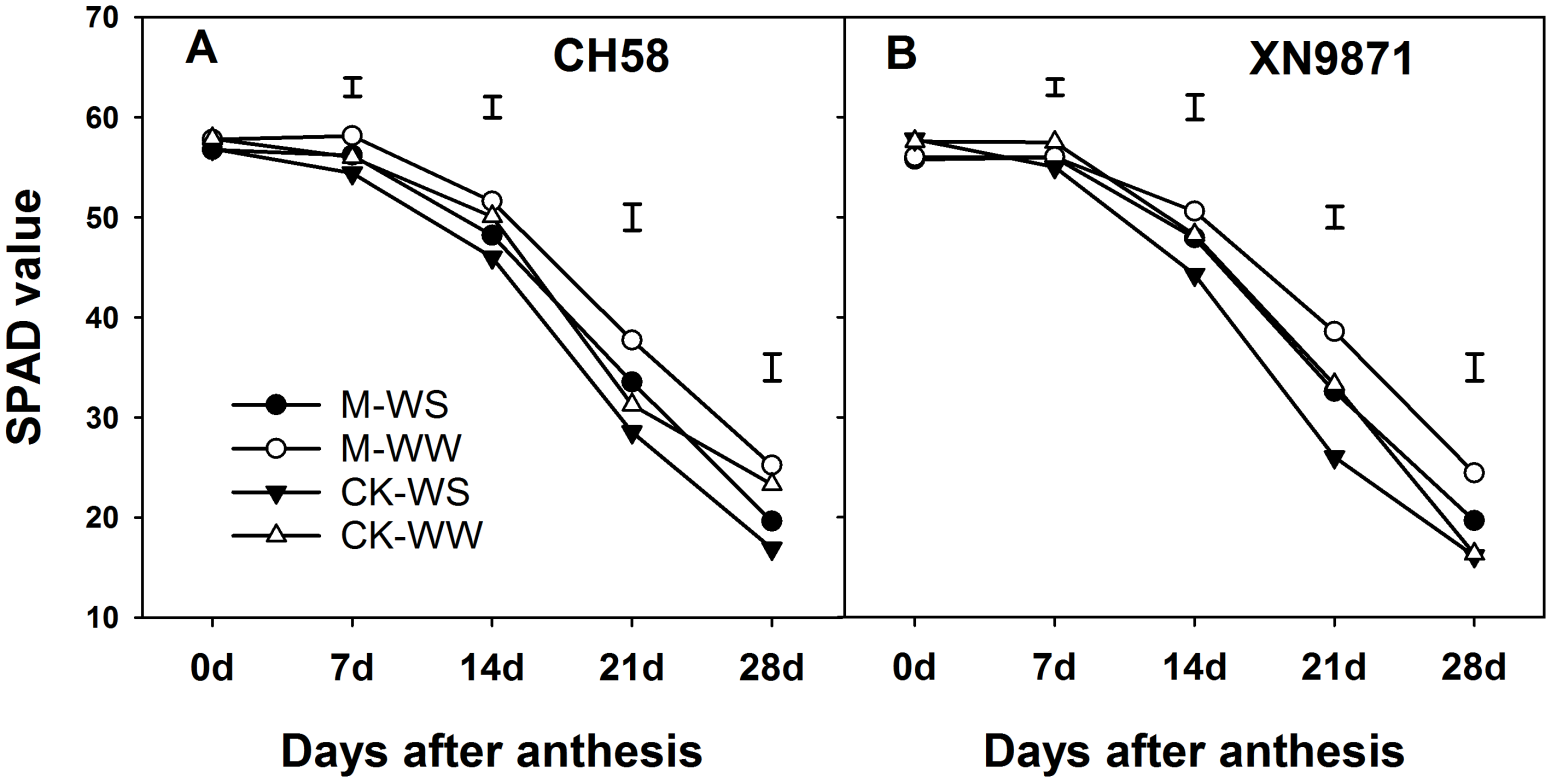
Changes in chlorophyll content in the flag leaf of two wheat cultivars, CH58 (A) and XN9871 (B) under organic fertilizer and different water regimes. CK: control; M: organic fertilizer; WS: water stress; WW: well-watered. Bars are LSD at *P*≤0.05. Effects of organic fertilizer on photosynthetic characteristics.

To investigate the differential leaf senescence following organic fertilizer application, we measured the changes in Fv/Fm and SPAD value during grain filling (Figs 3 and 4). There was no significant difference between cultivars in Fv/Fm and SPAD values during grain filling (*P*>0.05). Under WW, the Fv/Fm in CK decreased sharply from 14 d after anthesis in CH58 and 7 d in XN9871; under drought conditions, a marked change in the Fv/Fm of the CK treatment was noted 7 d after anthesis under both cultivars. Whereas a marked change in the Fv/Fm of the M treatment was noted until 14 d after anthesis under both water conditions. The WS treatment hastened the decline in Fv/Fm during 14–28 d after anthesis compared with WW, however, the M treatment had much higher Fv/Fm during 14–28 d after anthesis and obviously prolonged the duration of higher Fv/Fm compared with CK (Fig 3). Similarly, the SPAD value gradually declined after anthesis. A rapid reduction in SPAD value appeared at 7 d after anthesis under both water conditions. The SPAD value was decreased by WS compared with WW, whereas M treatment maintained higher values than CK during 14–28 d after anthesis (Fig 4).

**Fig 5:**
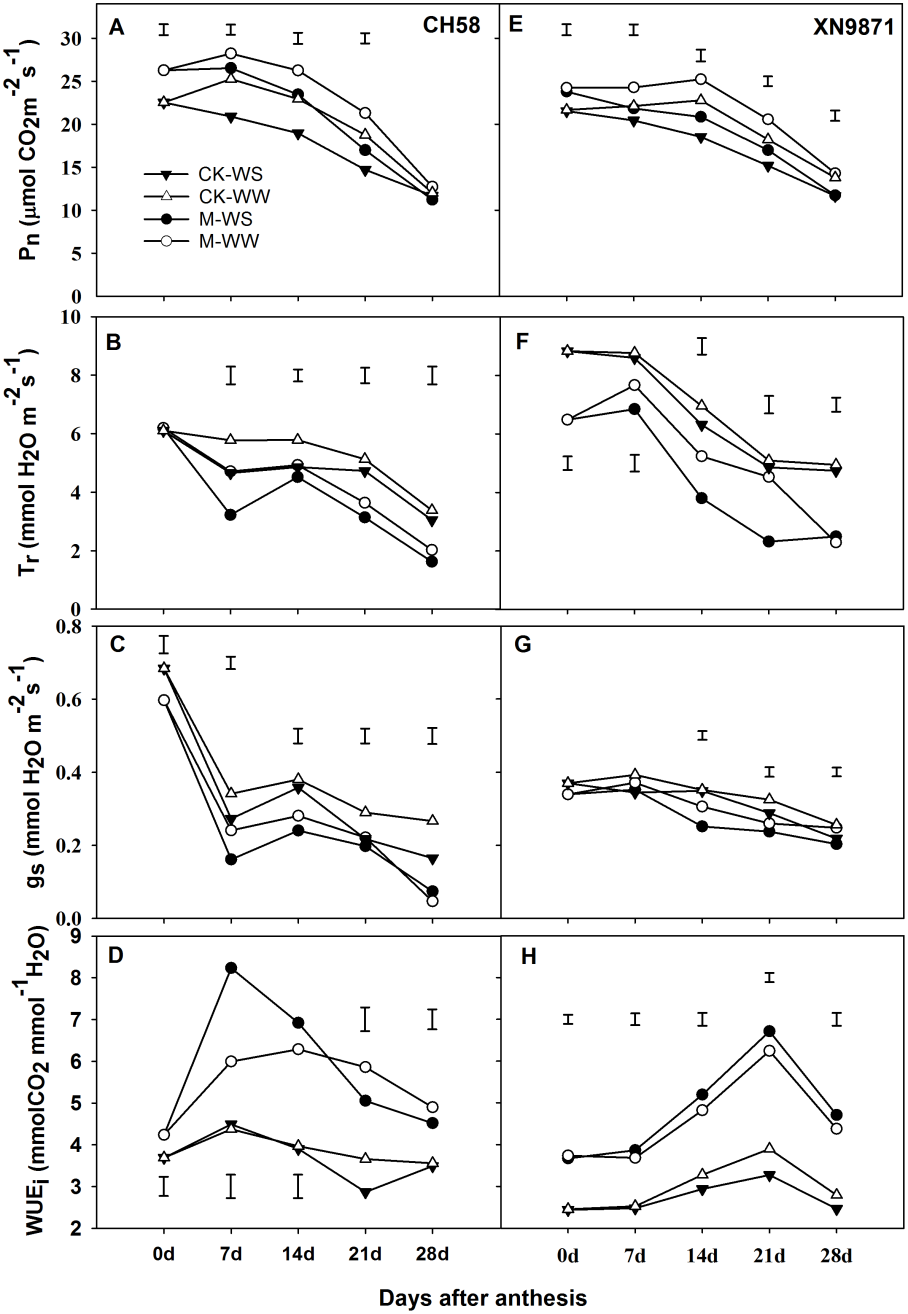
Changes in (A, E) net photosynthetic rate (P_n_), (B, F) transpiration rate (T_r_), (C, G) stomatal conductance (g_s_), and (D, H) water-use efficiency at the leaf level (WUE_i_) during the grain-filling stage of two cultivars, CH58 (A-D) and XN9871 (E-H) under organic fertilizer and two water regimes. Organic fertilizer: M; control: CK; WS: water stress; WW: well-watered. Bars are LSD at *P*≤0.05.

The changes in P_n_ during grain filling in the two cultivars (Fig 5) are similar to those in Fv/Fm and SPAD. The P_n_, g_s_, and T_r_ gradually declined after anthesis, and there was a significant difference in P_n_ between the M and CK treatments. Generally, the P_n_ in M treatment was much higher than in CK. For CH58, the P_n_ in M treatment was significantly higher than that in CK during 0–28 d after anthesis (Fig 5A); however, compared with CK, there was a significant difference in P_n_ during 0–21 d after anthesis in XN9871 under the M treatment (Fig 5E). There was a significant reduction in P_n_ until 14 d after anthesis in the WW treatment; however, a rapid reduction in P_n_ occurred 7 d after anthesis for both cultivars under the WS treatment. When WS was imposed, the average P_n_ throughout the grain-filling period was significantly reduced by 11.0 and 12.5% in CH58 and XN9871, respectively. The M treatment delayed leaf senescence and prolonged the duration of higher P_n_ under both water conditions. In contrast, the M treatment caused a noticeable decline in T_r_ and g_s_. Compared with CK, T_r_ was reduced by 6.4 and 24.2% in CH58 and XN9871, respectively, under the WW treatment and by 7.2 and 34.2%, respectively, under the WS treatment (Fig 5B and 5F). Similarly, the M treatment decreased g_s_ by 29.3 and 10.1% for CH58 and XN9871, respectively (*P*<0.05), under the WW condition and by 25.1 and 11.8%, respectively (*P*<0.05), under the WS condition, compared with CK (Fig 5C and 5G). Thus, transpiration efficiency, which is the WUE at the leaf level (WUE_i_), was significantly higher for the M treatment than for the CK treatment (Fig 5D and 5H). The difference in P_n_, T_r_ and g_s_ between cultivars might be associated with the different drought resistance and different air temperature and relative humidity (RH) at the same days after anthesis because of the different periods of anthesis.

### Effects of organic fertilizer on post-anthesis DM and N accumulation

The post-anthesis DM accumulation (PADMA) and post-anthesis N uptake (PANU) were affected by cultivars, CH58 had higher PADMA than XN9871 (+9.8%, *P*<0.05), but XN9871 had PANU (+45.0%, *P*<0.05) owing to its earlier flowering (Table 2). Averaged across both cultivars, WS markedly decreased PADMA and PANU by 36.0 and 53.0%, respectively (*P*<0.001). The M treatment increased significantly the PADMA and PANU by 51.5 and 80.4%, respectively, under WS and by 29.6 and 16.6%, respectively, under WW compared with CK. The F×W interaction was statistically significant for PANU; the C×W interaction was statistically significant for PADMA and PANU. However, the C×F interaction was not statistically significant for PANU (Table 2).

**Table 2:** Effects of organic fertilizer, water conditions and cultivars on DM accumulation and N uptake after anthesis and pre-anthesis DM and N remobilization and remobilization efficiency.

Treatments			DMR	DMRE	PADMA	PANU
Fertilizers	Water	Cultivars	(g pot^−1^)	(%)	(g pot^−1^)	(mg pot^−1^)
CK	WS	CH58	17.16	16.27	31.44	172.96
		XN9871	17.52	22.59	32.4	245.76
	WW	CH58	11.88	10.93	41.28	338.16
		XN9871	4.80	6.23	47.44	713.40
	Mean		12.84	14.00	38.14	367.57
M	WS	CH58	5.88	6.28	57.24	336.00
		XN9871	18.48	24.72	39.48	419.40
	WW	CH58	4.20	4.41	61.56	437.28
		XN9871	7.80	10.26	53.4	789.00
	Mean		9.09	11.42	52.92	495.42
LSD (0.05)			0.95	1.19	4.19	48.63
			Probability level of ANOVA			
C			**	ns	**	*
F			***	***	***	**
W			**	***	***	***
C×F			**	*	**	ns
F×W			**	ns	ns	**
C×W			**	ns	**	**
F×W×C			ns	ns	ns	ns

DMR: DM remobilization; DMRE: DM remobilization efficiency; PADMA: post-anthesis DM accumulation; PANU: post-anthesis N uptake; F: Fertilizers; C: Cultivars; W: Water; WS: water stress; WW: well-watered.

* indicated significant differences at *P* = 0.05 level

** indicated significant differences at *P* = 0.01 level

*** indicated significant differences at *P* = 0.001 level

ns: not significant.

### Effects of organic fertilizer pre-anthesis DM remobilization

Water stress clearly increased the DM remobilization (DMR) and DM remobilization efficiency (DMRE) by 105 and 119%, respectively, compared with WW (*P*<0.001) (Table 2). The DMR and DMRE were significantly lower under M treatment than CK under both water conditions. Averaged for both cultivars, M treatment significantly reduced the DMR by 30 and 28% and DMRE by 20 and 15% compared with CK under WS and WW, respectively. The F×W interaction was statistically significant for DMR; the C×W interaction was statistically significant for DMR. However, the C×F interaction was statistically significant for DMRE and DMR (Table 2).

### Effects of organic fertilizer on grain filling

**Fig 6:**
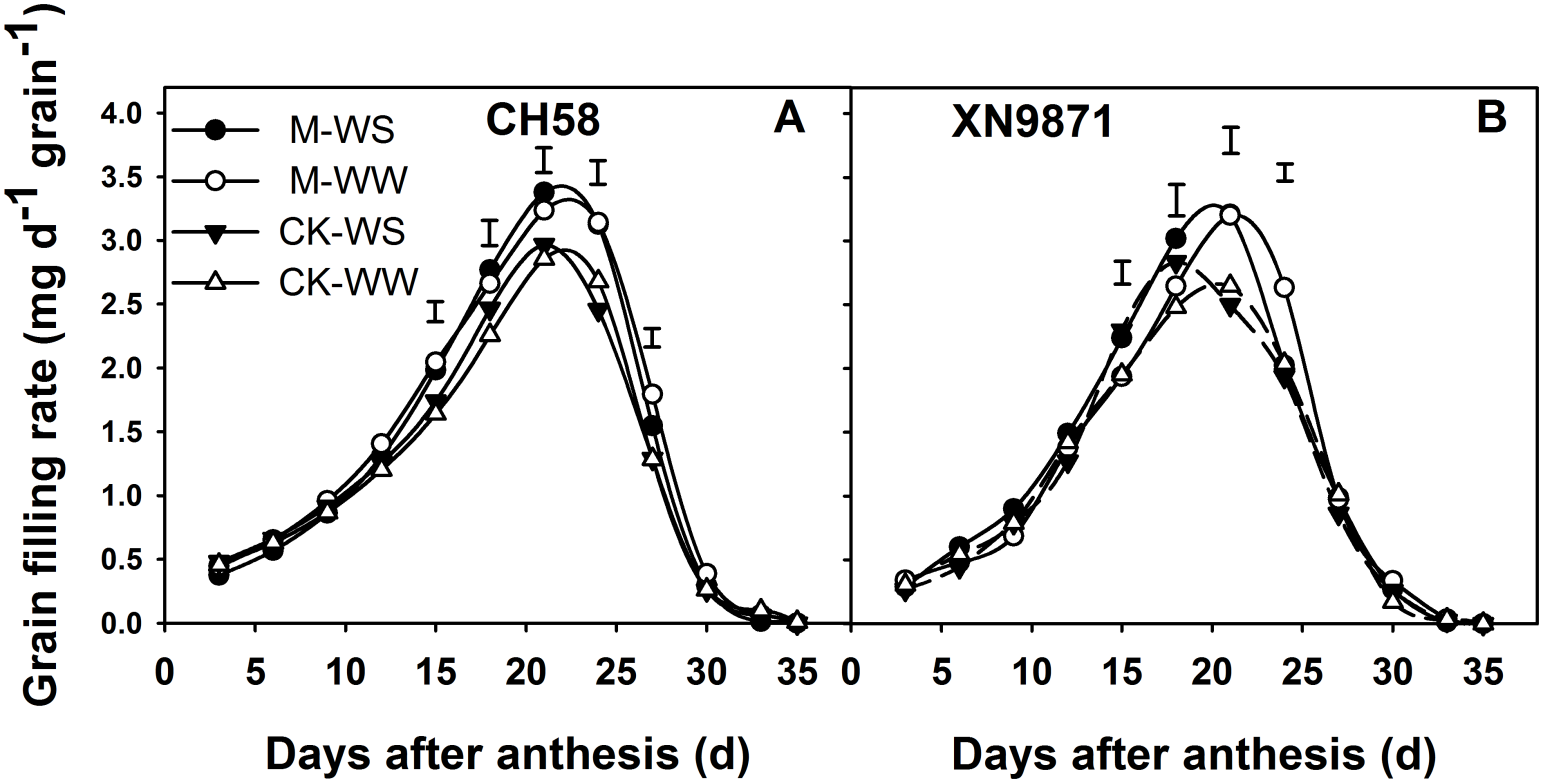
Changes in grain filling rate of two wheat cultivars, CH58 (A) and XN9871 (B) under organic fertilizer and different water regimes. The grain-filling rates were calculated according to the Richard equation [34]. CK: control; M: organic fertilizer; WS: water stress; WW: well-watered. Bars are LSD at *P*≤0.05.

**Fig 7:**
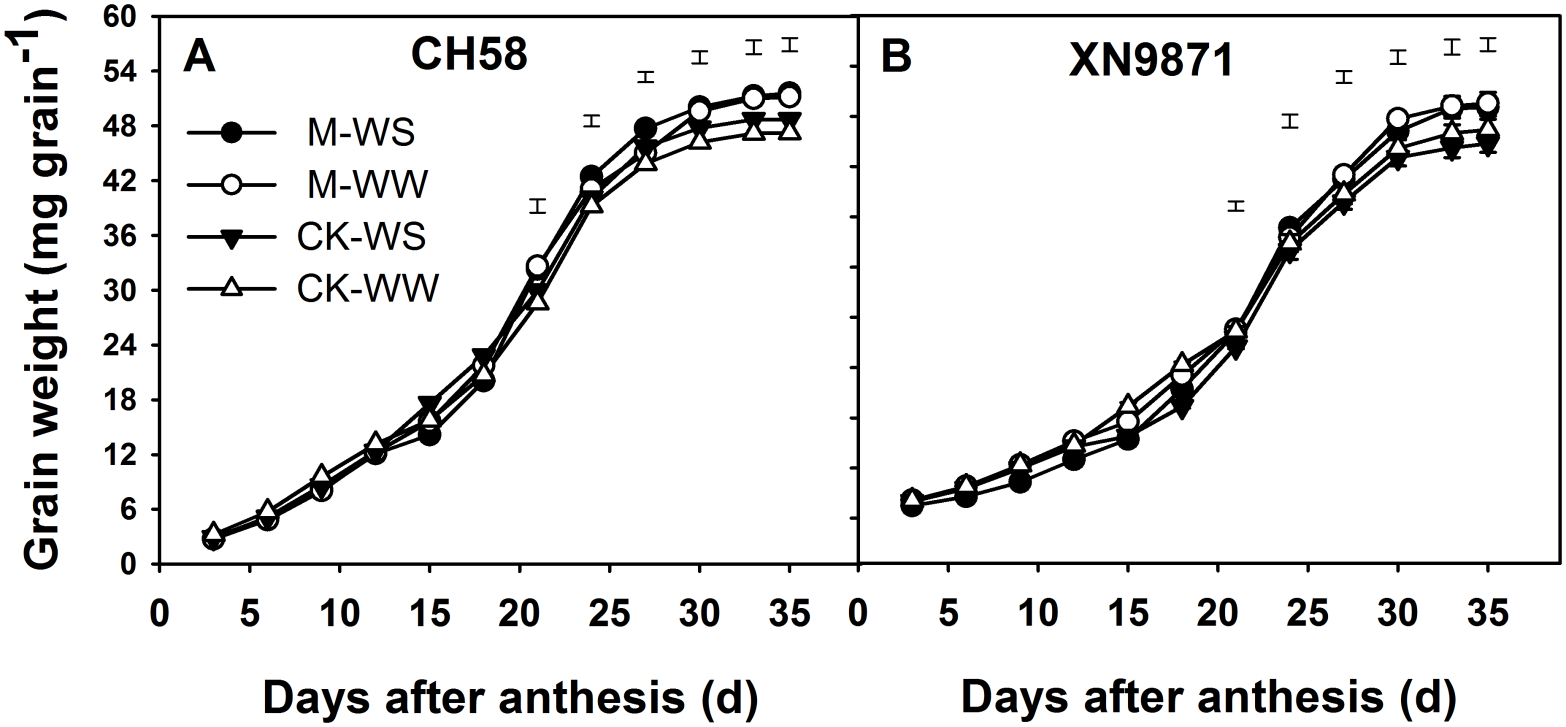
Changes in grain weight of two wheat cultivars, CH58 (A) and XN9871 (B) under organic fertilizer and different water regimes. CK: control; M: organic fertilizer; WS: water stress; WW: well-watered. Bars are LSD at *P*≤0.05.

The increases in grain weight and grain-filling rate of the wheat cultivars fitted into Richards’ growth equation are shown in Figs 6 and 7. The grain-filling rate was faster and the final grain weight higher under M than CK. In CH58, the grain-filling rate peaked at 21 d post-anthesis, with the time to peak rate slightly earlier in CK than M treatment (Fig 6A). Similarly, in XN9871, the grain-filling rate peaked at 21 d post-anthesis except for CK—WS treatment for which it was at 18 d (Fig 6B). Date showed that the final grain weight was positively correlated with the average grain filling rate during 15–27 d (CH58, XN9871; r = 0.971, *P* = 0.030, r = 0.942, *P* = 0.043, respectively). The WS shortened the time to peak grain-filling rate, however, M treatment substantially lengthened the time to maximum grain-filling rate compared with CK. In addition, the M treatment had a higher grain-filling rate than CK at the middle grain-filling stage, thus producing the heaviest grains (Fig 7A and 7B).

### Effects of organic fertilizer on biomass accumulation, grain yield and water use

Generally, the stem weight (StemWt), ear weight (EWt), and total biomass (TWt) at harvest were significantly higher under M treatment than CK (Table 3). The root/shoot ratio (R/S) was noticeably lower under M treatment. There were no significant differences in root weight (RWt) and leaf weight (LWt) between the M and CK treatments. The 1000-grain weight (TGW), grain number (GN), and yield were significantly higher in M treatment than in CK (+5.4, 11.8, and 18.9%, respectively). On average, the WS noticeably increased RWt and R/S by 14.2 and 34.4%, and reduced EWt, GN and yield by 19.5, 4.0 and 7.5%, respectively, compared with WW. However, LWt and StemWt were not markedly affected by WS. The LWt, StemWt, TWt, R/S, TGW, and HI differed between the varieties (*P*<0.05), but there was no significant difference in yield between CH58 and XN9871. In CH58, WS during grain filling increased TGW in both CK and M treatments compared with WW, whereas in XN9871, WS increased TGW in M treatment but decreased it in CK compared with WW. The HI was affected by organic fertilization; on average, M treatment increased HI by 1.5% compared with CK (*P*<0.05). Water stress during grain filling increased HI owing to increasing DMR and DMRE compared with WW (*P*<0.05). The W×F interactions was statistically significant for EWt, TWt, R/S and TGW but not for HI, RWt, LWt, GN, yield and StemWt. The C×F interactions was statistically significant for LWt and EWt. The W×F×C interactions was statistically significant with respect to R/S and TGW, in contrast to RWt, EWt, TWt, HI, LWt, StemWt, GN and yield (Table 3).

**Table 3:** Effects of organic fertilizer, water conditions and cultivars on root weight (RWt), leaf weight (LWt), stem weight (StemWt), ear weight (EWt), total dry biomass (TWt), 1000-grain weight (TGW), grain number per plant (GN), root/shoot ratio (R/S), grain yield per plant and harvest index (HI).

Treatments			RWt	LWt	StemWt	EWt	TWt	R/S	TGW	GN	Yield	HI
Fertilizers	Water	Cultivars	(g plant^−1^)						(g)	(plant^−1^)	(g plant^−1^)	
CK	WS	CH58	1.05	1.44	2.84	4.28	9.62	0.12	48.68	90.01	4.05	42.10
		XN9871	0.96	0.79	2.11	4.61	8.47	0.13	43.86	94.15	4.16	47.11
	WW	CH58	0.87	1.27	3.26	5.89	11.28	0.08	47.24	93.13	4.43	39.27
		XN9871	0.84	0.86	2.63	6.58	10.92	0.08	44.97	98.25	4.77	43.68
	Mean		0.93	1.09	2.71	5.34	10.07	0.10	46.19	93.89	4.35	43.04
M	WS	CH58	0.92	1.31	3.65	6.20	12.08	0.08	50.96	103.88	5.26	43.54
		XN9871	0.94	0.94	3.05	5.83	10.76	0.10	48.29	101.39	4.83	44.89
	WW	CH58	0.82	1.14	3.88	7.07	12.91	0.07	50.49	108.17	5.48	42.45
		XN9871	0.86	0.94	2.55	6.46	10.81	0.09	45.02	106.55	5.10	47.18
	Mean		0.88	1.08	3.28	6.39	11.63	0.08	48.69	105.00	5.17	44.51
LSD (0.05)			0.17	0.23	0.51	0.66	1.00	0.006	1.33	8.71	0.37	1.20
			Probability level of ANOVA									
W			***	ns	ns	*	**	***	***	*	***	*
F			ns	ns	**	***	***	***	***	***	***	*
C			ns	***	***	ns	**	*	***	ns	ns	***
W×F			ns	ns	ns	***	***	***	***	ns	ns	ns
W×C			ns	ns	ns	*	ns	ns	ns	ns	ns	ns
F×C			ns	*	ns	*	ns	ns	ns	ns	***	ns
W×F×C			ns	ns	ns	ns	ns	**	***	ns	ns	ns

F: Fertilizers; C: Cultivars; W: Water; WS: water stress; WW: well-watered.

* indicated significant differences at *P* = 0.05 level

** indicated significant differences at *P* = 0.01 level

*** indicated significant differences at *P* = 0.001 level

ns: not significant.

On average, the water consumption rate (WCR) and total water consumption per plant (TWC) were lower in M treatment than in CK (WCR, TWC; *P*<0.05, *P*>0.05, respectively). Under WS, the TWC and WCR did not differ significantly between M and CK treatments, but the M treatment significantly decreased TWC by 9% under WW. Also, the M treatment significantly increased WUE_B_ under WS and WW by 51 and 45% compared with CK, respectively (Table 4). On average, the M treatment increased WUE_g_ under WS and WW by 25 and 23%, respectively, compared with CK (*P*<0.05). The C×W and C×F interactions were statistically significant for WUE_B_ but were not significant for WUE_g_. The W×F×C interactions was not statistically significant for WUE_B_ or WUE_g_. Results indicated that the responses of WUEg to M treatment depended on water and cultivars.

**Table 4:** Effects of organic fertilizer, water conditions and cultivars on total water consumption at grain filling stage (TWC), water use efficiency for biomass yield (WUE_B_) and grain yield (WUEg), and water consumption rate (WCR) at grain filling stage.

Treatments			TWC	WUE_B_	WUE_g_	WCR
Fertilizers	Water	Cultivars	(kg plant^−1^)	(g kg^−1^)	(g kg^−1^)	(g d^−1^ plant^−1^)
CK	WS	CH58	1.19	2.20	1.78	34.04
		XN9871	1.36	1.86	1.71	38.75
	WW	CH58	1.92	1.80	1.48	54.73
		XN9871	2.15	1.84	1.48	61.34
	Mean		1.65	1.92	1.61	47.22
M	WS	CH58	1.35	3.69	2.21	38.60
		XN9871	1.21	2.44	2.16	34.45
	WW	CH58	1.76	3.06	1.80	50.15
		XN9871	1.68	2.20	1.85	47.86
	Mean		1.50	2.85	2.01	42.76
LSD (0.05)			0.19	0.21	0.15	0.75
			Probability level of ANOVA			
W			***	***	***	***
F			ns	***	***	*
C			ns	**	*	ns
W×F			ns	ns	ns	ns
C×W			ns	**	ns	*
C×F			ns	**	ns	ns
W×F×C			ns	ns	ns	ns

F: Fertilizers; C: Cultivars; W: Water; WS: water stress; WW: well-watered.

* indicated significant differences at *P* = 0.05 level

** indicated significant differences at *P* = 0.01 level

*** indicated significant differences at *P* = 0.001 level

ns: not significant.

## Discussion

### Effects of organic fertilizer on dry matter accumulation and remobilization, and their relationships with yield and WUE

The accumulation and remobilization of DM during grain filling are important processes for yield formation and WUE. Application of organic fertilizer application significantly increased grain yield and WUE in many studies [10,14,36–38], however, little attention was paid to the effects of organic fertilizer on the accumulation, partitioning and remobilization of DM, and their relationships with yield formation and WUE. In this study, the M treatment favored the allocation of photosynthate to aboveground plant parts, this is important for improving yield and WUE, and supports previous reports [17,39,40]. To improve the capacity of plants for water capture, RWt was higher under WS than WW, and resulted in greater R/S than WW as reported previously [31]. WS stimulated plants to allocate a greater proportion of photosynthate to the ears through enhancing DMR and DMRE, in agreement with previous studies [4,41–44]. However, under M treatment, an increase in post-anthesis DM accumulation allowed a lower use of pre-anthesis DM reserves, consistent with previous study [44]. In M treatment, an increase in the N assimilated after anthesis enhanced post-anthesis DM accumulation and grain yield, indicating greater N assimilated after anthesis was beneficial for improving yield, which is in agreement with previous studies [45,46].

Numerous studies have shown that drought stress reduced grain yield owing to reductions in kernel growth rate [47] or by shortening the duration of grain filling [48]. In our study, WS shortened the duration of grain filling but increased the grain-filling rate, mainly because of the significant increases in DMR and DMRE, consistent with previous study [44]. However, WS during grain filling greatly reduced grain yield owing to a great decrease in post-anthesis DM accumulation, consistent with the previous studies [31,49]. In contrast, the yield improvement in M treatment resulted from large increases in post-anthesis DM accumulation and post-anthesis N uptake, which agree with the previous studies [37,45]. Most importantly, although the M treatment greatly improved DM accumulation and grain yield, it had a similar TWC to CK under both water conditions, which is in agreement with the results from other studies [10,13,14]. Consequently, the M treatment resulted in a large increase in WUE_B_ compared with CK. We speculate that there was a physiological response involved in the regulation of WUE.

### Physiological responses of wheat to improved WUE with organic fertilizer

The LRWC is defined as the percentage of actual leaf water content to saturated leaf water content and indicates the water status of plants; the EWLR and WRC can indicate the cuticular transpiration (non-stomatal transpiration) and anti-transpiration abilities of inherent leaf structures [50]. In the present study, there was no notable difference in LRWC of the two cultivars between fertilizer treatments—consistent with a previous study in maize [51]. However, M treatment significantly affected the Ψ_w_, EWLR and WRC, indicating better water relations under WS. The changes in Ψ_w_, EWLR and WRC under M treatment not only maintained the leaf water content but also enhanced the P_n_ and WUE under WS, consistent with previous studies [4,50,51].

Photosynthesis is usually reduced when plants suffer from drought stress, thus excessive excitation energy may induce photoinhibition of photosynthesis and even oxidative damage to the photosynthetic apparatus [51]. The content of MDA has been used as an index to qualify the degree of membrane lipid peroxidation [24]. To cope with oxidative damage under extremely adverse conditions, plants have developed an antioxidant defense system that includes the antioxidant enzymes SOD, ascorbate peroxidase (APX), POD, and catalase (CAT) [24,52,53]. In our study, WS increased the MDA content compared with WW, in agreement with previous studies [24,26,54–56]. However, M treatment had lower MDA content under WS, achieved by regulating the activities of SOD and POD. The changes in activities of SOD and POD in M treatment were beneficial towards increasing drought resistance and maintaining high P_n_ under WS, which is consistent with the findings of previous studies [16,24,26,57–59].

Senescence occurs during the last phase of ontogeny of a leaf, and it is characterized by a marked decline in the assimilatory capacities, which was associated with massive degradation of cellular proteins [60]. Although senescence remobilization contributes nitrogen and other nutrients for seed growth, the concomitant drop in photosynthetic activity limits the yield of several important crops [61]. Our results indicated that WS remarkably decreased post-anthesis N uptake—a result consistent with a previous study [31], and induced early leaf senescence, shortening the duration of higher P_n_ during grain filling. This ultimately decreased post-anthesis DM accumulation, grain yield and WUE, in agreement with results from other studies [31,61–63]. However, an increase in post-anthesis N uptake under M treatment increased directly grain yield and WUE due to increasing photosynthetic capacity and delaying leaf senescence, in agreement with previous studies [2,45,64].

In cereals, the photosynthesis during the post-anthesis period is the major carbohydrate source for grain filling and yield formation [42,65]. The post-anthesis DM accumulation can contribute to 80–95% of the final grain yield under non-stress conditions [66]. The increase in P_n_ has the potential to increase crop yield, provided other constraints do not become limiting [67–69]. Our results indicated that M treatment had larger increases in post-anthesis DM accumulation, yield and WUE_B_ under WS, mainly because of an increase in photosynthetic capacity and decrease in T_r_ and g_s_, which is in agreement with results from previous studies [4,17,40,65–71]. These rsults suggest that the organic fertilizer enabled the plants to use water more efficiently under water stress. Under water stress, the increase in photosynthetic capacity and decrease in T_r_ in M treatment were important for sustainable agricultural production and food security in the semiarid areas.

However, we also found clear differences in TGW between the cultivars for CK and M treatments under WS. These contrasting results may be related to the difference in drought resistance between CH58 and XN9871. CH58 has a stronger drought resistance than XN9871; therefore, under WS it showed a smaller reduction in PADMA than XN9871. One the other hand, WS enhanced the remobilization of pre-anthesis carbon reserves and reduced the GN compared with WW; thus, for CH58, WS increased the TGW whether in CK or M treatment. However, because XN9871 in CK under WS suffered a larger reduction in PADMA than CH58, the WS decreased TGW in CK compared with WW. In contrast, because of the better water relations and less damage under WS in plants treated with M, the XN9871 had a smaller reduction in photosynthetic capacity and PADMA. At the same time, XN9871 exhibited a higher reduction in GN in the M treatment than in CK under WS. Consequently, in XN9871, WS increased TGW under M treatment compared with WW, consistent with previous studies [67–70]. Results suggests that the improved WUE under M treatment resulted from two factors: large increases in grain yield due to increasing post-anthesis DM accumulation, via improved drought resistance under WS and delayed leaf senescence after anthesis; and improved WUE_i_ from large increases in P_n_ and reductions in T_r_.

In conclusion, the M treatment increased photosynthetic capacity and delayed leaf senescence by increasing post-anthesis N uptake, thus favoring greater P_n_ after anthesis. Moreover, the M treatment reduced g_s_ and T_r_; it especially maintained better water relations and caused less damage under WS. These changes not only increased post-anthesis DM accumulation, grain number per plant and 1000-grain weight, but also, relatively, decreased leaf water loss. Therefore, M treatment increased grain yield, WUE_B_ and WUE_g_ relative to CK. Our results suggest that the organic fertilizer enabled the plants to use water more efficiently under water stress.

## Supporting information

S1 Dataset Contains data on POD activity, SOD activity, MDA content, maximum efficiency of PSII photochemistry (Fv/Fm), chlorophyll content, photosynthesis, ear dry weight, ear N content and grain filling rate in flag leaves, ears and grains. (XLSX)

S2 Dataset Contains data on weather conditions, leaf water potential, relative leaf water content, water retention capacity, excised-leaf water loss rate, post-anthesis dry matter accumulation, pre-anthesis dry matter remobilization, post-anthesis N uptake and pre-anthesis N remobilization during grain filling stage. (XLSX)

S3 Dataset Contains data on root weight, leaf weight, stem weight, ear weight, total dry biomass, 1000-grain weight, grain number per plant, root/shoot ratio, grain yield per plant and harvest index at harvest. (XLSX)

## Abbreviations

DM: Dry matter
DMR: Dry matter remobilization
DMRE: Dry matter remobilization efficiency
EWLR: Excised-leaf water loss rate
Fv/Fm: Maximal quantum yield of PSII photochemistry
GN: Grain number per plant
HI: Harvest index
LRWC: Leaf relative water content
M: Manure treatment
MDA: Malondialdehyde
N: Nitrogen
NR: N remobilization
NRE: N remobilization efficiency
PADMA: Post-anthesis DM accumulation
PANU: Post-anthesis N uptake
POD: Peroxidase
R/S: Root to shoot ratio
SOD: Superoxide dismutase
TGW: 1000-grain weight
TWC: Total water consumption during grain filling stage
WCR: Water consumption rate during grain filling stage
WRC: Water retention capacity
WUE_B_: Water use effificiency for biomass yield
WUE_g_: Water use effificiency for grain yield
WUE_i_: Water use efficiency at the leaf level
